# Cross-Sectional Serosurvey of Companion Animals Housed with SARS-CoV-2–Infected Owners, Italy

**DOI:** 10.3201/eid2707.203314

**Published:** 2021-07

**Authors:** Barbara Colitti, Luigi Bertolotti, Alessandro Mannelli, Gianmarco Ferrara, Andrea Vercelli, Andrea Grassi, Claudio Trentin, Saverio Paltrinieri, Chiara Nogarol, Nicola Decaro, Emiliana Brocchi, Sergio Rosati

**Affiliations:** University of Turin, Turin, Italy (B. Colitti, L. Bertolotti, A. Mannelli, S. Rosati);; University of Naples, Naples, Italy (G. Ferrara);; Clinica veterinaria Città di Torino, Turin, Italy (A. Vercelli);; I-Vet srl Laboratorio di Analisi Veterinarie, Flero, Italy (A. Grassi);; AUSL Valle d'Aosta, Aosta, Italy (C. Trentin);; University of Milan, Milan, Italy (S. Paltrinieri); IN3Diagnostic, Turin (C. Nogarol);; University of Bari, Bari, Italy (N. Decaro);; Istituto Zooprofilattico Sperimentale della Lombardia e dell’Emilia Romagna, Brescia, Italy (E. Brocchi)

**Keywords:** respiratory infections, severe acute respiratory syndrome coronavirus 2, SARS-CoV-2, SARS, COVID-19, coronavirus disease, zoonoses, viruses, coronavirus, cats, dogs, One Health

## Abstract

We conducted a serologic survey among dogs and cats in Italy to detect antibodies against severe acute respiratory syndrome virus 2 (SARS-CoV-2). We found that SARS-CoV-2 seroprevalence was higher among cats (16.2%) than dogs (2.3%). In addition, seroprevalence was higher among animals living in close contact with SARS-CoV-2–positive owners.

After emerging in Wuhan, China, in December 2019, coronavirus disease (COVID-19), caused by severe acute respiratory syndrome coronavirus 2 (SARS-CoV-2), rapidly became a serious threat to human health worldwide (*1*–*3*). Italy has experienced one of the highest rates of human deaths in the world (*4*).

Questions concerning the role of companion animals in the COVID-19 pandemic arose after a dog in Hong Kong reportedly tested positive for SARS-CoV-2 (*5*). In addition, the World Organisation for Animal Health defined COVID-19 as an emerging disease in animals and began promoting surveys on the prevalence of SARS-CoV-2 infections among animals (*6*). In this context, serologic tests are essential for rapid and accurate screening of animal populations. 

Few studies have been conducted to clarify the effects domestic animals have in sustaining the SARS-CoV-2 transmission cycle (*5*,*7*–*9*; Q. Zhang et al., unpub. data, https://www.biorxiv.org/content/10.1101/2020.04.01.021196v1). Because Italy suffered high COVID-19 incidence rates and the country has >32 million companion animals, health authorities were interested in examining virus transmission between humans and animals. We conducted a cross-sectional serologic survey among domestic dogs and cats in Italy to identify a possible association between SARS-CoV-2 infection in humans and animals. We used serologic tests to detect specific antibodies from animals living in close contact with SARS-CoV-2–positive human patients. 

## The Study

Blood was collected from pets during routine activities performed by veterinary practitioners, who shared serum samples with us. Owners provided written consent for research purposes. We used 198 samples, 130 from dogs and 68 from cats, collected during the March–June 2020 COVID-19 epidemic in Italy and 100 serum samples, 65 from dogs and from 35 cats, collected in different regions of Italy before 2019 as prepandemic controls.

A recombinant antigen corresponding to the nucleocapsid (N) protein of SARS-CoV-2 has been expressed in human embryonic kidney 293T cells, which have been used to develop Eradikit COVID19-IgG (IN3diagnostic, https://www.in3diagnostic.com) a sensitive and specific ELISA to detect SARS-CoV-2 antibodies in human serum samples. However, our initial attempts to validate the specificity of this ELISA on pet serum samples were unsuccessful. We switched the reaction from solid-phase to solution-phase kinetics using the same antigen and the specificity improved. Thus, we used a novel immunoassay, xMAP (Luminex Corp., https://www.luminexcorp.com), which is based on paramagnetic beads. We developed a flow cytometry-based system and applied it to serum samples from cats and dogs.

To define the test’s specificity, we analyzed pre-epidemic samples and expressed results as the mean fluorescence intensity (MFI) ratio of a sample-to-positive control. On the basis of reactivity distribution, we set the discriminative cutoff to 40% MFI of the positive control. Using these specifications, we recorded diagnostic specificities of 96.5% (95% CI 87.9%–99.6%) for dog serum and 100.0% (95% CI 90.0%–100.0%) for cat serum.

Our choice of the viral N protein might raise concern because dogs and cats are susceptible to species-specific coronaviruses. The amino acid similarity between SARS-CoV-2 and the canine betacoronavirus, canine respiratory coronavirus, is slightly higher than canine and feline alphacoronaviruses (*10*), which could explain the suboptimal specificity obtained in pre-epidemic dog samples. In fact, 2 serum samples gave reactivity slightly over the cutoff value. However, when potential cross-reactivity of the N protein between SARS-CoV-2 and endemic human coronaviruses was evaluated, no reactivity was shown against human coronaviruses 229E, OC43, HKU1, or NL63 by western blot or ELISA (*11*), suggesting that similar results might be expected from phylogenetically related feline and canine coronaviruses (*12*).

**Figure 1:**
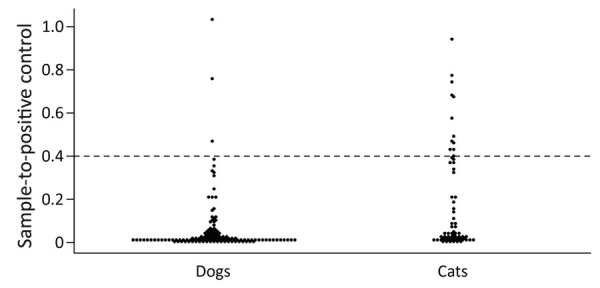
Distribution of sample-to-positive severe acute respiratory syndrome coronavirus 2 serology results among dogs and cats, Italy, March–June 2020. Horizontal dashed line represents the positive-negative discriminatory cutoff.

Among samples collected during the epidemic period, 7.1% (14/198) tested positive by the serologic test. In all, 147 animals (54 cats and 93 dogs) lived in households with SARS-CoV-2–positive owners. All 14 seropositive animals lived with SARS-CoV-2–infected owners and percent positivity was greater among cats than dogs (Table 1; Figure 1). Among animals living with SARS-CoV-2–infected owners, 20.4% (11/54) of cats and 3.2% (3/93) of dogs were seropositive. 

**Table 1:** Seropositivity among cats and dogs tested for antibodies against severe acute respiratory syndrome coronavirus 2, Italy, March–June 2020

**Level of analysis**	% Positivity among pets
**Individual animal**	
Owners’ status*	
** Infected, n = 147**	9.5
** Not tested, n = 49**	0
** Animal species**	
** Cat, n = 68**	16.2
** Dog, n = 130**	2.3
** Living conditions†**	
** Indoor, n = 87**	12.6
** Outdoor, n = 51**	3.9
**Households tested, n = 156‡**	
** Owners’ status**	
** Infected, n = 111**	10.8
** Not tested, n = 45**	0
** Cats tested in the household**	
** Yes, n = 51**	19.6
** No, n = 105**	1.9
** Dogs tested in the household**	
** Yes, n = 114**	2.6
** No, n = 42**	21.4


*Information on owners’ status was missing from 1 cat and 1 dog. Infected means positive molecular tests for the detection of SARS-Cov-2 in >1 owner in a household. Percent positivity at the household level was based on finding >1 seropositive animal. †Information on pets’ living conditions was missing for 43 dogs and 17 cats. ‡Households were considered positive if >1 pet was serologically positive. Households were divided into those in which >1 cat was tested and those in which >1 dog was tested.

**Figure 2:**
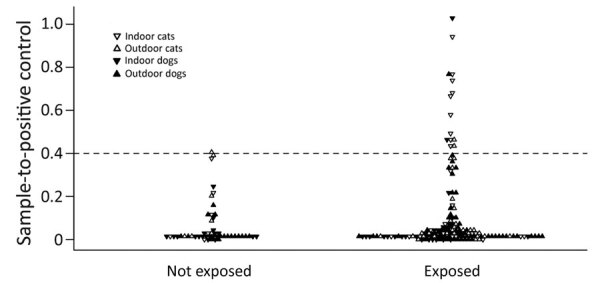
Distribution of sample-to-positive severe acute respiratory syndrome coronavirus 2 serology results among dogs and cats exposed and unexposed to positive owners, Italy, March–June 2020. Horizontal dashed line represents the positive-negative discriminatory cutoff.

Exact logistic regression analysis indicated a positive association between owners’ infections and seropositivity in individual animals, after adjusting for animal species (Figure 2). The odds of finding >1 seropositive animal in a household were positively associated with owners’ infection and with an increasing number of tested cats (Table 2; Appendix). The association with owner infection was only statistically significant based on a 1-tailed hypothesis, whether the outcome was measured at the animal or household level.

**Table 2:** Results of multivariable exact logistic regression of the association between severe acute respiratory syndrome coronavirus 2 seropositivity among cats and dogs and infected owners, Italy, March–June 2020

**Level of analysis**	Odds ratio (95% CI)	p value*
**Individual animal**		
** Owners’ status**	6.1† (0.97–∞)	0.055
** Cat vs. dog**	7.6 (1.9–44.4)	0.002
**Household**		
** Owners’ status**	5.8† (0.9–∞)	0.068
** No. tested cats**	2.5 (1.3–5.2)	0.008
*p value based on 2-tailed test. †Median unbiased estimate.		


Using exact logistic regression, we noted the percent of positive results was greater for animals living indoors only than for animals with access to the outside (odds ratio 3.4, 95% CI 0.71–35.9), but the association was not statistically significant (p = 0.15). Because information on living conditions was missing for 60 animals, we did not include this factor in the exact logistic regression analysis (Tables 1, 2).

We found the proportion of serologic positivity increased with increasing length of exposure. We recorded the first SARS-CoV-2–positive animals 10 days after owners’ diagnoses and all 14 seropositive cases were classified as positive after >54 days of exposure (Appendix).

Among 5/14 positive animals, owners reported that their pets experienced clinical signs concurrent with the owner’s COVID-19 illness. In particular, a 10-year-old male dog showed respiratory signs (cough, sneezing) after which he had vomiting and diarrhea in concomitance with the onset of the owners’ symptoms; a 1-year-old dog showed mild respiratory signs characterized by cough and sneezing; a 12-year-old female cat showed respiratory signs characterized by rhinitis with abundant nasal discharge. Furthermore, a 13-year-old female cat was hospitalized for a brachial cephalic thrombosis and a 3-year-old male cat was hospitalized for interstitial pneumonia. Of note, 3 asymptomatic SARS-CoV-2–positive cats belonged to a single-family cluster in which both owners tested positive and hospitalized.

## Conclusions

We detected antibodies against the SARS-CoV-2 N protein in pets living with SARS-CoV-2–infected owners. A higher percentage of feline samples tested positive, confirming a higher susceptibility and prevalence in cats than in dogs reported in previous experiments (*10*,*13*). The susceptibility of cats to SARS-related human coronaviruses also was reported in 2003 when a study confirmed that cats were susceptible to infection and could transmit the virus to other in-contact animals (*14*). The association between seropositivity in animals and the confirmed SARS-CoV-2 infection in >1 of the animal’s owners was statistically significant (p<0.05) based on a 1-sided test assuming the owner’s infection could not reasonably exert a protective effect on pets’ infection. 

We could not draw conclusions concerning the direction of viral transmission in this cross-sectional study. Nevertheless, our results, coupled with the direction of the association between seropositivity and length of exposure to an infected owner and living indoors, suggest that the development of antibodies in pets might be a consequence of viral transmission from their owners. Additional studies with more statistical power could confirm these relationships.

Based on our results, future studies should focus on overcoming test limitations by improving specificity in dog serum samples through detailed epitope mapping of the N protein. Additional studies also should examine routes and risk factors for transmission of SARS-CoV-2 from infected persons to susceptible pets and the potential role of pets in the COVID-19 pandemic. Clinical and pathological consequences of SARS-CoV-2 infection in cats and dogs also warrant further research.

In conclusion, our study on companion animals housed with SARS-CoV-2–infected humans confirms the susceptibility of domestic cats under natural exposure. Our data statistically support other findings that cats are more susceptible than dogs and that living in contact with >1 SARS-CoV-2–infected person increases the risk for infection in pets. These results justify the need to adopt control measures in SARS-CoV-2–infected pet owners to reduce viral transmission to their companion animals.

Appendix Additional information on a serosurvey of companion animals housed with SARS-CoV-2–positive owners, Italy.
