# Supplementary material for: Cross-Sectional Serosurvey of Companion Animals Housed with SARS-CoV-2–Infected Owners, Italy
Source: Emerg Infect Dis. 2021 Jul;27(7):1919–22. doi: 10.3201/eid2707.203314 (PMC8237875; doi:10.3201/eid2707.203314)
Supplement: Appendix — Additional information on a serosurvey of companion animals housed with SARS-CoV-2–positive owners, Italy. [file 20-3314-Techapp-s1.pdf]

Article DOI: https://doi/.org/10.3201/eid2707.203314

Cross-Sectional Serosurvey of Companion Animals Housed with SARS-CoV-2–Infected Owners, Italy

Appendix

Sampled Animals

The pre-epidemic panel consisted of serum samples collected from 65 dogs and 35 cats in different regions of Italy before 2019. During the March–June 2020 coronavirus disease (COVID-19) epidemic in Italy, serum samples of 198 animals from different regions of Italy were collected, including samples from 68 cats and 130 dogs. Among them, 54 cats and 93 dogs had been housed in close contact with COVID-19–affected patients whose infections were confirmed by detection methods for severe acute respiratory syndrome coronavirus 2 (SARS-CoV-2). The remaining serum samples were from animals with no exposure or no confirmed exposure to SARS-CoV-2, although samples were collected in areas with confirmed viral circulation.

Written consent and the answers to a questionnaire were obtained from all pet owners, at the time of blood collection from animals. Owners were asked whether they had been tested for SARS-CoV-2 by nucleic acid tests and whether the test was positive. The following information was collected for each animal: name, sex, age, lifestyle (whether indoor or outdoor), and presence of chronic diseases. Time and duration of exposure to the infected owners were recorded for each animal, corresponding to the time between the first positive swab test in the owner and the end of possible animal exposure, such as when the owner was hospitalized or had a negative swab test. To avoid unnecessary health risks for veterinary practitioners, all samples were collected after the owners quarantined and had a negative SARS-CoV-2 test. Moreover, because animal serum samples were collected after their owners' quarantine was over, no animals were expected to be symptomatic. Only information about animal clinical signs that occurred concurrently with owners' illness were collected.

Serology

SARS-CoV-2 xMAP Assay

To set up the xMAP (Luminex Corp., https://www.luminexcorp.com) SARS-CoV-2 nucleocapsid (N) bead-based immunoassay, IN3diagnostic (https://www.in3diagnostic.com) provided a SARS-CoV-2 recombinant N. Paramagnetic Bio-Plex Pro COOH Beads (Bio-Rad, http://www.bio-rad.com) were coupled by carbodiimide chemistry with bovine beta-casein previously modified with Sulfo-NHS-LC-Biotin (Thermo Fisher Scientific, https://www.thermofisher.com). Then, recombinant N expressed in fusion with monomeric streptavidin was incubated with beads for 1 hour under agitation. After 2 washes, beads were resuspended in phosphate-buffered saline (PBS) and 0.05% sodium azide (NaN_3) and stored at 4°C protected from light until use.

Serum samples were diluted 1:200 in 100 µL of assay buffer (PBS, 0.05% Tween 20, and 0.05% NaN_3), in 96-well flat-bottom plates, mixed with 1,750 beads/well and incubated for 1 h at room temperature on a plate shaker set to 850 rpm, protected from light. The plate was washed twice by using a magnetic plate separator. As a secondary antibody, protein G (for dog serum) or protein A (for cat serum) was conjugated with phycoeritrin (PG-PE for dogs or PA-PE for cats; Moss Bio, https://mossbio.com) according to manufacturer's instructions. The resulting conjugate was used at 1:500 for dog serum and 1:250 for cat serum in assay buffer. Plates were incubated on a plate shaker for 30 min at room temperature, protected from light and then washed twice by using magnetic plate separator, as mentioned. Finally, 100 µL of wash buffer was added to each well and plates were read with the BioPlex200 (Bio-Rad) platform.

The median fluorescence intensity (MFI) was recorded for each sample and for negative and positive controls. Positive controls were represented by a hyperimmune serum produced in a goat immunized with the recombinant N antigen provided by the manufacturer in the Eradikit COVID19-IgG (IN3diagnostic) ELISA and was used for normalizing interplate experiments.

Statistical Analysis

We tested pre-epidemic serum samples and used these reactivities to assess the assay cutoff. We calculated the mean reactivity plus 3× the SD value and compared this with the reactivity of the positive control.

We performed statistical analysis to evaluate the role of different epidemiologic conditions in increasing the risk for positive results in pets. In detail, we evaluated the length of animal exposure to SARS-CoV-2–infected human patients, the animal species, the living habits, and the first day of exposure.

We tested the association between owner's confirmed infection and the odds of positive serologic test in animals by using the exlogistic command in Stata version 15.1 to perform exact logistic regression (*1*,*2*). This approach was justified by a quasicomplete separation of our results due to the frequency of 0 positive results among unexposed animals that belonged to untested owners. At the individual animal level, we adjusted for species, by including a dichotomous predictor, comparing cats versus dogs as the reference species.

Animals from 156 households were tested. Due to the heterogeneous and generally small number of animals tested in each household, we did not use methods to account for nonindependence of test results on animals belonging to the same household, such as generalized estimating equations or random intercept logistic regression (*3*). As an alternative, we analyzed data at the household level. Accordingly, we considered a household positive if $\geq$1 tested animal was positive to antibodies against SARS-CoV-2; otherwise, the household was considered negative. Only 1 animal was tested in 8/12 (66.7%) positive households; 3 animals tested positive in 1 household; and $\leq$5 animals were tested in another household, and 1 tested positive. Among 144 negative households, only 1 animal was tested in 120 (83.3%); $\leq$4 animals were tested in 2 households. We analyzed the association between the owners' infection status and animals' serologic results at the household level by exact logistic regression (*1*) after adjusting for the number of tested animals, and for animal species, by including the number of tested cats and tested dogs as 2 separate predictors. We then eliminated the number of dogs, as a predictor, from the final model, since it was not associated with the outcome and its exclusion did not affect parameters for the other predictors. Pets' living conditions, indoors or outdoors, were not included in the exact logistic regression analysis with other predictors because information on this risk factor was missing for 60 animals.

Finally, we evaluated the time of exposure of each animal by considering the contact period between the animal and its owner when the SARS-CoV-2–positive owner was considered potentially infectious. We assumed the exposure period started on the date of the owner's first positive molecular diagnostic result and it ended when molecular diagnostic tests were negative or the owner left the house for hospitalization. Cumulative distribution of

positive animal samples was evaluated by dividing the exposure time in bins of 10-day units and used as categorical values for the description of serologic responses of tested animals.

References

1. StataCorp. Stata: release 15. Statistical software. College Station, TX: StataCorp LLC. 2017. https://www.stata.com/manuals15/u.pdf

2. Hirji KF, Mehta CR, Patel NR. Computing distributions for exact logistic regression. J Am Stat Assoc. 1987;82:1110–7. https://doi.org/10.1080/01621459.1987.10478547

3. Molenberghs G, Verbeke G. Models for discrete longitudinal data. New York: Springer Science+Business; 2005.

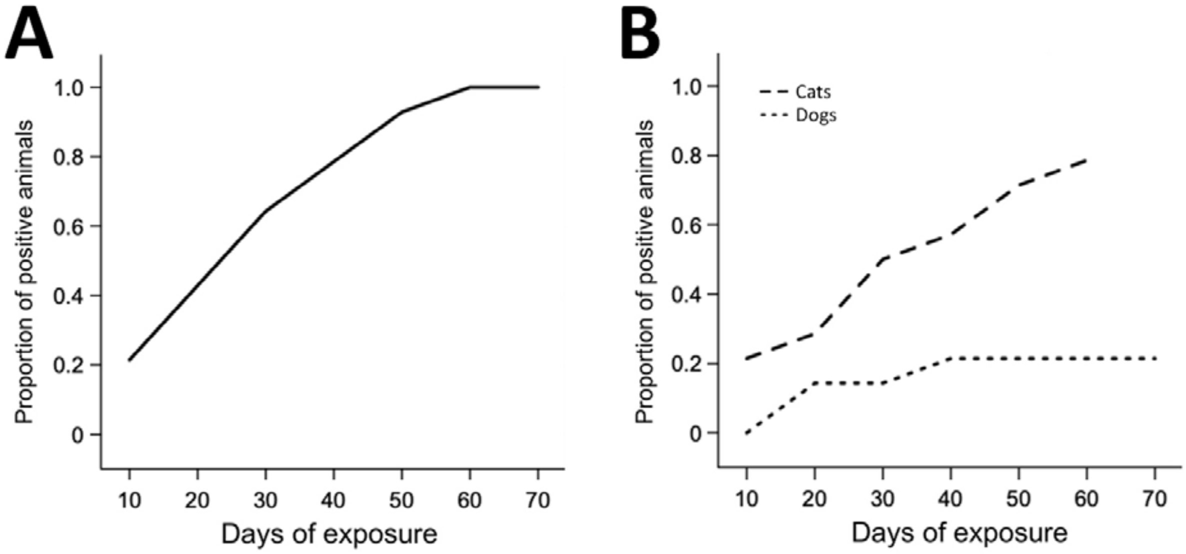

Appendix Figure. Cumulative distribution of SARS-CoV-2–positive serum samples among tested animals over time, Italy. The proportion of positive animals is correlated with the length of time animals were exposed to owners who tested positive for SARS-CoV-2. A) Cumulative positive results among all animals tested. B) Cumulative distribution among cat samples and dog samples. SARS-CoV-2, severe acute respiratory syndrome coronavirus 2.
